# The Alumni Association of the Chicago Medical College

**Published:** 1875-04-01

**Authors:** S. A. McWilliams

**Affiliations:** 125 Eighteenth street, Chicago


### THE ALUMNI ASSOCIATION OF THE CHICAGO MEDICAL COLLEGE.

THE Alumni Association of the Chicago Medical College met in the parlors of the Tremont House, March 15th, at 8 p. m.

Dr. F. C. Winslow presided.

A larger number than usual were present. Communications of importance were received from Dr. John M. Woodworth, of the United States Marine Hospital Service; from Dr. John Sundburg, of Galveston, Texas, and from Dr. George H. Fuller, now at Fort Hall Agency, Ross Fork, Idaho.

Dr. Wm. E. Quine resigned his position of Necrologist, and Dr. S. A. McWilliams that of Secretary and Treasurer.

Both of these resignations were accepted.

The Committee on Entertainment now announced supper to be ready, and invited all present to partake. This part of the programme was a decided success.

After supper, Professor H. A. Johnson was invited to address the Alumni, which he did in his usual happy and eloquent manner.

The Committee on Nominations made the following report, which was received and adopted: S. A. McWilliams, M.D., President, Class '66, Chicago, Illinois; H. H. Clark, M.D., First Vice-President, Class '70, McGregor, Iowa; H. D. Ensign, M.D., Second Vice-President, Class '75, of Chicago Marine Hospital; M. P. Hatfield, M.D., Necrologist, Class '72, 1604 State street, Chicago, Ill.; D. A. K. Steele, M.D., Secretary and Treasurer, Class '73, 883 State street, Chicago, Ill., to whom all communications should be addressed.

The Committee on Entertainment for the ensuing year are as follows: The President and Secretary, with Drs. Bond, Earle, and Hutchinson.

It is to be hoped that our Alumni will present at our next annual meeting many scientific and valuable papers.

S. A. McWilliams.
125 Eighteenth street, Chicago.

